# Advising patients on the use of non-alcoholic beverages that mirror alcohol

**DOI:** 10.1016/j.pmedr.2024.102888

**Published:** 2024-09-18

**Authors:** Molly A. Bowdring, Geoffrey W. Rutledge, Judith J. Prochaska

**Affiliations:** a Stanford University School of Medicine, Stanford Prevention Research Center, 3180 Porter Drive, Palo Alto, CA 94304, USA; b Stanford University School of Medicine, Department of Psychiatry and Behavioral Sciences, 401 Quarry Road, Stanford, CA 94304, USA; c HealthTap, 2465 Latham Street Third Floor, Mountain View, CA 94040, USA

**Keywords:** Alcohol-free, Zero-alcohol, NA drinks, Hepatology, Perinatal, Psychiatry, Internal medicine

## Abstract

• Non-alcoholic beverages that mirror alcohol have potential benefits and risks.
• Providers are currently offering varied health guidance on use of these beverages.
• Research is needed to yield consensus on non-alcoholic beverage use health effects.

While non-alcoholic beverages (NABs) mirroring alcoholic beverages have been around for decades, the World Health Organization reports striking growth in use of non-alcoholic beers, wines, spirits, and mocktails in recent years (World Health Organization, 2023). Containing <0.5 % alcohol and often marketed as a healthy alternative to full-strength alcoholic beverages, research on NABs’ health impacts is lacking (World Health Organization, 2023). Providers are nonetheless likely to encounter patients asking for guidance on NAB use safety.

To examine how providers address NAB use, we reviewed publicly-available data from HealthTap – a digital health service that hosts a repository of anonymous patient questions covering a range of health topics and responses from US-licensed doctors (deemed exempt by Stanford University’s IRB). We searched for questions related to NABs posed 2011–2024. Search terms included: (drinks; beverages; malt drinks; beer; wine; cocktail; mocktail; liquor) AND (non alcoholic; non-alcoholic; nonalcoholic; alcohol-free; alcohol free; zero-alcohol; zero alcohol; zero-proof; 0-proof; 0 proof; spirit-free; spirit free; no-alcohol; no alcohol). Of 40 questions identified, three topics emerged with most inquiries – NAB use with liver disease (10), with psychotropic medication use (8), and during the perinatal period (5). It is expected that conditions wherein alcohol use is not recommended – or at least questionable (in the case of psychotropic medication use) – would be most likely to spur patient uncertainty about NAB safety. Table 1 highlights examples of patient questions and provider guidance, with some encouraging NAB abstinence and others welcoming moderate consumption or expressing no concern. The varied provider guidance is unsurprising, given limited data on health impacts of NABs. We elaborate first on the potential relation between NAB and alcohol use as a general consideration for providers, then expand on specific topics raised in patient questions, outlining important concerns and unanswered questions.

**Table 1:** Examples of patient questions about non-alcoholic beverage use and HealthTap provider responses collected from 2011 to 2024.

**Topic**	**Patient Questions**	**Provider Responses**	**Guidance** • Abstain • Moderate • No Concern
***Liver Disease***	Can someone with a compromised liver (steatotic liver disease, cirrhosis) drink non-alcoholic beverages?	Abstinence is generally recommended. However your case may be more or less so. Talk with your doc.	Abstain
		Good for you in stopping alcohol! Beer legally can be labeled and sold as non-alcoholic as long as they contain no more than 0.5 % alcohol. Most nonalcoholic beers are near that limit, e.g. 0.4–0.5 %. Probably 1–2 beers nonalcoholic beers daily is OK, but no more.	Moderate
		Certainly a person with cirrhosis can have non-alcoholic beverages.	No concern

***Psychotropic Medication***	Can someone taking benzodiazepine (e.g., clonazepam, lorazepam, alprazolam) drink non-alcoholic beverages?	Adding any amount of alcohol to any amount of medication, especially those which suppress nervous system function (e.g. clonazepam) is never something a doctor would ever find acceptable. Most important reason? It's dangerous. Consequences such as respiratory depression, coma, and death occur daily.	Abstain
		If you have an underlying alcohol problem, clonazepam itself may be a problem for you, but the taste and smell of beer can be a trigger to move from the non-alcoholic variety (which still has about 0.5 % alcohol) to something stronger. If there is no underlying alcohol issue, the tiny amount of alcohol in the beer will not interact seriously with clonazepam in most people.	Abstain if alcohol use disorder; No concern of no alcohol use disorder
		Depends on the dose of Lorazepam and amount of non-alcoholic beer.	Moderate
		Yes there is no reason why not.	No concern
	Can someone taking an selective serotonin reuptake inhibitor (e.g., sertraline, paroxetine) drink non-alcoholic beverages?	The recommendation is NO alcohol with medication.	Abstain
		If you have an issue, just limit the beer. Even if non-alcoholic.	Moderate
		Alcohol together with a medication like sertraline may cause dizziness, drowsiness, and difficulty concentrating. Alcohol-free variants are not a problem − they may be consumed by a person taking sertraline.	No concern

***Perinatal Period***	Can a pregnant person drink non-alcoholic beverages?	Avoid alcohol completely during pregnancy for the health of your baby and yourself. Even though studies in Denmark showed no effect on growth in low level consumption of alcohol, it is well known alcohol is related to prematurity, birth defects, and other issues. How is the medical provider who encouraged you to drink going to respond if that happens to your baby? Follow the American Academy of Pediatrics advice.	Abstain
		Enjoy. Don’t drink too many though, it could cause excess weight gain.	Moderate
		It is ok. In some countries, pregnant ladies drink even more without significant problems.	No concern
	Can you drink non-alcoholic beer while breastfeeding to increase breast milk?	If there is ABSOLUTELY NO alcohol in the product I could consider it harmless. The near beers that have low amounts in the product are still a danger. Any alcohol will cross easily into the breast milk & one of its byproducts is formaldehyde, sometimes used as an embalming agent.	No concern of 0.0 %; Abstain otherwise

*Note.* Question and response wording were edited lightly for clarity.

## 1 The potential influence of NABs on alcohol use

While NAB alcohol content is minimal – the legally-permitted alcohol level in NABs is comparable to what naturally occurs in some fruit juices (Gorgus et al., 2016) – we encourage consideration about the potential impact of NABs on alcohol use via cue-reactivity. The similarity of NABs in appearance and taste to alcohol are not only what makes them an appealing substitute but also what may confer risk for alcohol craving and full-strength alcohol consumption (Bowdring et al., 2024). Alcohol research has revealed that alcohol cues (e.g., images of alcohol or others drinking) prompt physiological cue reactivity, craving, and alcohol consumptions (Schacht et al., 2013, Witteman et al., 2015, Jones et al., 2013). Such cues are also necessarily present in NABs, which are designed to mimic the look of alcohol.

Because not everyone is highly cue-reactive, NABs may be an acceptable substitute to alcohol for some. Further research is needed to clarify whether and under what conditions alcohol cues in NABs prompt craving and subsequent alcohol use and, conversely, for whom NABs satisfy craving and support reduction of alcohol use (Bowdring et al., 2024, Yoshimoto et al., 2023). Assessing alcohol use history and perceived effect of NABs on alcohol craving to guide patients on NAB use may prove beneficial, particularly when working with individuals for whom alcohol use is especially risky (e.g., people with liver disease, people taking psychotropic medications, pregnant patients, people with alcohol use disorder).

## 2 Patients with liver disease

Mixed responses from providers about NAB use among people with liver disease mirror the mixed guidance offered in the few scientific articles on this topic. Some studies have outlined potential benefits of NABs in individuals with liver disease. A 2016 consensus document on the effects of moderate beer consumption on health and disease from an international panel of independent scientists stated that NABs can be a useful alternative among individuals for whom alcohol consumption is contraindicated, including people with liver disease (de Gaetano et al., 2016). In addition, a recent 8-week clinical trial randomized patients (*N* = 43) with cirrhosis to either an intervention group that received non-alcoholic beer or a control group assigned to consume water (both groups also received exercise and diet interventions) (Macías-Rodríguez et al., 2020). Patients who received non-alcoholic beer demonstrated greater improvements than the control group in endothelial functioning, nutrition, and quality of life. These clinical trial findings are encouraging but preliminary.

Nevertheless, NABs may present some unique risks in patients with liver disease. Important to consider is a published case study of a patient with end-stage liver disease and history of alcohol use disorder, in which consumption of 9 non-alcoholic beers across one evening and morning resulted in a blood alcohol concentration of 0.06g/dL (DiMartini and Rao, 1999). The authors highlight that compromised liver functioning impedes oxidation of ethanol by the liver and can yield elevated blood alcohol levels; they propose that complete abstinence from alcohol (including NABs) may be best practice for individuals with liver disease. The American Association for the Study of Liver Disease states that there is no safe level of alcohol use for individuals with liver disease (Bertha et al., 2021). Whether that guideline should be interpreted to include NABs remains to be determined. Further studies are needed to elucidate the safety of NAB use among patients with liver disease.

## 3 Patients taking psychotropic medications

There are a variety of psychotropic medications – patient questions about NAB use with benzodiazepines and selective serotonin reuptake inhibitors (SSRIs) were most commonly raised. Alcohol use is discouraged among people taking benzodiazepines, as these are sedative-hypnotics for which even moderate amounts of alcohol use (1–2 drinks) can amplify sedative effects (e.g., drowsiness) via the central nervous system (Weathermon and Crabb, 1999). SSRIs are widely used antidepressants that have a better safety profile than benzodiazepines and are not nearly as sedating. While research demonstrating serious interactions with alcohol is limited, clinical case reports indicate risk for increased alcohol craving, sensitivity to alcohol, and blackouts while taking SSRIs (Weathermon and Crabb, 1999, Menkes and Herxheimer, 2014).

Although the legal limit of NAB alcohol content is low, potential for cue-reactivity to transition NAB consumers to full-strength alcohol use would be a concern for patients taking SSRIs or benzodiazepines. Further research is needed to delineate the safety of NABs among patients taking specific psychotropic medications.

## 4 Patients in the perinatal period

The Centers for Disease Control and Prevention and the American College of Obstetricians and Gynecologists emphasize that no amount of alcohol is safe during pregnancy (CDC, 2023, American College of Obstetricians and Gynecologists, 2024). These entities have not made specific statements about NAB consumption among pregnant people. Though data are scarce, prior work suggests that people believe NABs to be helpful for blending in socially while pregnant (Skagerström et al., 2015). Market research, however, suggests that people are uncertain about whether NABs are safe during pregnancy and find NAB labeling ambiguous (Corfe et al., 2020). One study conducted in 2010 tested 45 NAB products and found that 29 % of products had higher alcohol content than what was noted on their labels, with six products labeled as either alcohol-free (denoting 0.0 % ABV) and non-alcoholic (denoting <.5 % ABV) observed to contain >1 % ABV (Goh et al., 2010). It is unclear to what extent discordance between NAB labels and actual alcohol content presents in today’s marketplace. Updated evaluations of alcohol content and user-friendly labels are needed to facilitate informed decision making by individuals who are pregnant and consumers more broadly.

Regarding NAB use while breastfeeding, one study found that moderate consumption of 0.0 % beer yields increased antioxidant capacity in the breastmilk and lower oxidative stress in the breastfeeding individual (Codoñer-Franch et al., 2013). Another study tested risk of ethanol exposure to the child via breastmilk by having 15 breastfeeding individuals each consume four non-alcoholic beers in one hour. Alcohol was not detected in the majority of breastmilk samples tested, with trace amounts (maximum of 0.0002g/dL) detected in just two samples (Schneider et al., 2013). While these authors suggested that NAB use is safe (Schneider et al., 2013), others have encouraged delaying breastfeeding to reduce risk of ethanol exposure to the child (Adiong et al., 2014). Additional research is needed to inform guidance on acceptable quantity and pace of NAB consumption among individuals who are breastfeeding.

## 5 Conclusion

NAB use is booming and providers are likely to encounter patients with diverse clinical profiles who consume these beverages. We highlighted a few clinical conditions in which questions about NAB use most often arose within the context of a single digital health service. HealthTap providers who chose to respond to NAB questions may not be representative of providers at large, and public responses may differ from how providers would respond in private clinical encounters. Further, HealthTap patients may not be representative of the broader population and their alcohol use history was not assessed. Thus, the clinical data utilized in this report likely did not capture all presenting conditions warranting guidance on NAB use. For example, high carbohydrate content of some NABs (e.g., sugary mocktails) may have a negative impact on diabetes. While NABs may help reduce alcohol use among some people who consume alcohol (Yoshimoto et al., 2023), the potential for alcohol use reduction vs. cue reactivity with NABs for patients with high-risk alcohol use warrants investigation (as does assessment of how addiction providers currently advise patients) (Bowdring et al., 2024). NAB use among underage youth also is largely unregulated and worth assessing in practice (Miller et al., 2022, Bowdring and Prochaska, 2024). As the research base develops, providers are encouraged to stay abreast of the health effects of NAB use across medical conditions to facilitate evidence-based clinical care. Clinical practice governing bodies and public health professionals will also play a crucial role in disseminating knowledge on the potential benefits vs. risks of NABs. There is a need for formal committees to conduct a review of the evidence and develop practice guidelines regarding the use of NABs in various clinical conditions.

## 6 Disclosure of Funding and Conflicts of Interest

This publication was made possible by an NHLBI-funded postdoctoral fellowship to MAB (5T32HL161270-02). NHLBI had no role in study design; collection, analysis, or interpretation of data; writing the report; or the decision to submit the report for publication. Outside of this research, MAB has consulted for a technology company aimed at helping people to reduce their alcohol use. GWR is employed by HealthTap. The authors have no other financial disclosures.

## CRediT authorship contribution statement

**Molly A. Bowdring:** Writing – review & editing, Writing – original draft, Methodology, Conceptualization. **Geoffrey W. Rutledge:** Writing – review & editing, Resources, Data curation, Conceptualization. **Judith J. Prochaska:** Writing – review & editing, Methodology, Conceptualization.

## Declaration of competing interest

The authors declare the following financial interests/personal relationships which may be considered as potential competing interests: Molly A. Bowdring reports financial support was provided by National Heart Lung and Blood Institute. Geoffrey W. Rutledge reports financial support was provided by HealthTap. Molly A. Bowdring reports a relationship with Pivot that includes: consulting or advisory. If there are other authors, they declare that they have no known competing financial interests or personal relationships that could have appeared to influence the work reported in this paper.

## Data Availability

Data will be made available on request.
